# Adolescent-onset cannabis use and parenting young children: an investigation of differential effectiveness of a digital parenting intervention

**DOI:** 10.3389/frcha.2024.1392541

**Published:** 2024-06-18

**Authors:** Katherine A. Hails, Anna Cecilia McWhirter, Audrey C. B. Sileci, Elizabeth A. Stormshak

**Affiliations:** Prevention Science Institute, University of Oregon, Eugene, OR, United States

**Keywords:** parenting, cannabis, adolescent substance use, digital health, early childhood

## Abstract

**Introduction:**

There is scant empirical work on associations between current and past cannabis use and parenting skills in parents of young children. As recreational cannabis use is now legal in nearly half of states in the U.S., cannabis use is becoming more ubiquitous.

**Methods:**

In the current study, parents of toddler and pre-school age children were randomly assigned to participate in an app-based parenting skills program that included telehealth coaching (Family Check-Up Online; FCU-O), with a focus on parenting in the context of substance use. We aimed to test associations between adolescent-onset and current cannabis use and parent mental health and parenting skills, as well as whether effects of the FCU-O on parent mental health outcomes varied as a function of past cannabis use. Participants were 356 parents of children ages 1.5–5 participating in a randomized controlled trial of the FCU-O. Parents screened into the study if they reported current or past substance misuse or current depressive symptoms. After completing a baseline assessment, parents were randomly assigned to the FCU-O or control group and completed a follow-up assessment 3 months later. Parents retrospectively reported on the age when they initially used substances, as well as their current use.

**Results:**

After accounting for current cannabis use, adolescent-onset cannabis use was significantly associated with higher symptoms of anxiety and depression, but not with parenting skills. Adolescent-onset cannabis use was found to significantly moderate the effect of the FCU-O on parents' anxiety symptoms. Specifically, the FCU-O was particularly effective in reducing anxiety symptoms for parents with adolescent-onset regular cannabis use, after accounting for current cannabis use.

**Discussion:**

Adolescent-onset regular cannabis use may be a risk factor for later mental health challenges in parents of children under 5. An app-based parenting intervention may be particularly helpful in reducing symptoms of anxiety for parents who used cannabis regularly as adolescents. The findings have significant implications for the prevention of multigenerational risk for substance use and mental health challenges.

## 1 Introduction

Cannabis use among adolescents and young adults in the United States has steadily increased over the past decade (1–3), likely attributable to the changing landscape of recreational cannabis legalization (4). This increase in cannabis use is potentially concerning as cannabis directly affects the brain, particularly executive functioning and areas responsible for memory, learning, attention, and decision-making, as well as coordination, emotion regulation, and reaction time (5–7). Given robust associations between cannabis use and psychosocial functioning (1), and increasing societal emphasis on disrupting intergenerational patterns of substance use (8), it is not surprising that there is growing interest in the effects of parental cannabis use on parenting and child development among researchers, practitioners, and policymakers (9, 10).

There are few empirical studies of how parental cannabis use may affect parenting skills and behaviors, particularly in parents of toddler and preschool-age children. It is essential to explore parental cannabis use in the context of parenting young children, given the importance of parent-child relationships during this critical developmental period (11) and potential substance-related disruptions to caregiving quality (12). In the few studies that have examined cannabis use in relation to parenting, researchers have primarily focused on negative parenting behaviors, including abuse and neglect. Researchers have found that cannabis use is associated with negative parenting practices in parents of children age 1.5–5 (13), higher rates of physical abuse, corporal punishment, and nonviolent discipline in parents of children under the age of 12 (14, 15), and child abuse and neglect in parents referred for drug treatment or child neglect (14). Far fewer studies have tested relationships between cannabis use and other aspects of parenting, particularly among parents of toddlers and preschoolers. One study of parents of adolescent children found that parental cannabis use disorder was associated with low levels of positive parenting (16). The relative lack of focus on associations between cannabis use and positive parenting, particularly among parents of young children, is a notable omission. Positive parenting strategies and interventions that work to strengthen the parent-child relationship help to prevent child maltreatment (17) and are associated with greater child self-regulation, a protective factor for adolescent risk-taking behaviors, including substance use (18).

In addition to understanding effects of concurrent cannabis use on parenting behaviors, it is also essential to determine how parents' cannabis use in adolescence, a critical period of neurological development (19), may impact their parenting years later. Theoretically, adolescents may be at heightened risk of experiencing adverse effects of cannabis due to their ongoing brain development (6, 7, 20). Moreover, adolescence is thought to be an especially vulnerable time for the development of substance use disorders. Adolescent cannabis users are more likely to develop cannabis use disorder than adults who use cannabis (21), and they also tend to experience more severe symptoms, including psychosis (22). Therefore, although there is a theoretical basis to suggest that adolescent-onset cannabis use may have a detrimental effect on parenting skills, even after accounting for current cannabis use, this has not been previously tested. More research is needed to better understand the relationship between early-onset cannabis use and later parenting practices.

In addition to being a sensitive period for the development of substance use disorders, adolescence is also the stage during which the most common mental health disorders, including anxiety and depression, tend to emerge (23). Importantly, cannabis use is associated with mental health concerns including symptoms of depression and anxiety, which are known to negatively impact parenting skills (24, 25). Depression has been found to predict subsequent weekly cannabis use after controlling for other substance use (26). Moreover, youth with more chronic or severe forms of depression during their early adolescent years are at elevated risk for developing a cannabis use disorder compared with youth with less severe symptoms (27). With regard to anxiety, a recent systematic review and meta-analysis found strong evidence for a significant relationship between adolescent cannabis use and the exacerbation of anxiety symptoms later in life (28), consistent with prior studies finding longitudinal symptoms of persistent anxiety among adolescent cannabis users (29). Although it remains unclear whether cannabis use increases depression and anxiety, whether symptoms of anxiety and depression drive increased cannabis use, or whether there are reciprocal effects on each, research indicates that adolescent-onset regular cannabis users are at increased risk of depressive symptoms compared with adult-onset users (30). Given the pernicious effects of depression and anxiety on parenting and child development (24, 25), it is important to understand the potential effects of both adolescent-onset and concurrent cannabis use on the psychological wellbeing of parents of young children.

Considering the potential risk for both parenting and mental health challenges among parents with current or past cannabis use, preventive parenting interventions may be especially important for this population. The Family Check-Up Online (FCU-O), adapted from the original Family Check-Up (FCU), is a brief digital health intervention focused on parenting skills and support (31). The original FCU is an evidence-based program that has been found to improve parenting skills (32), reduce child behavioral and emotional problems (33–35), and improve parents' mental health and wellbeing (36, 37). The FCU-O was developed as a telehealth model to allow for wide-scale dissemination of parenting strategies for parents at risk for substance use, especially for families with limited access to services such as in rural areas (31), and includes both a mobile app and telehealth coaching. The FCU-O has been associated with improvements in parent well-being (reduced anxiety, depression, and stress), parenting skills, co-parenting, parental confidence and self-efficacy, and a reduction in child emotional problems (38).^1^ The original FCU model has been found to be especially effective for parents experiencing distress, mental health challenges, and fewer resources (39). However, it is unknown whether the effectiveness of the FCU or the FCU-O is strengthened or weakened by a parent's history of or current substance use. Very few studies have compared the efficacy of parenting interventions for parents who are substance-involved with those who do not use substances (40).

### 1.1 Current study

Early onset of cannabis use in adolescence can lead to increased and more severe use in adulthood and has been linked to greater mental health and cognitive challenges (20, 21). Use of cannabis may additionally affect parenting skills (13), which has implications for the future substance use of children of cannabis users (41). The current study sought to explore whether adolescent onset of cannabis use, independent of current use, is associated with current parenting skills and mental health. We hypothesized that early-onset cannabis use in adolescence would be associated with greater anxiety and depression, and fewer parenting skills.

Considering the need to better understand and generate further hypotheses around adolescent cannabis use and future parenting, we also sought to test whether the efficacy of the FCU-O in improving anxiety and depression symptoms varies depending on parents' history of adolescent-onset cannabis use. Given the exploratory nature of this aim and a lack of prior research in this area, we do not specify a directional hypothesis.

## 2 Materials and methods

### 2.1 Participants

Participants are the primary caregivers (i.e., parent or legal guardian) (*N = *356) of children aged 1.5–5 years. Many recruitment strategies were used to recruit caregivers, including partnerships with community agencies and in-person events, as well as advertising on social media. To be eligible for participation in the study, caregivers had to endorse any one of the following: binge drinking or any recreational drug use (including cannabis) in the past year, lifetime history of opioid misuse, or endorsement of depressive symptoms on the Patient Health Questionnaire-2 [PHQ-2; (42)]. Parents also had to be living with their child at least 50% of the time, have a smart phone with text messaging capability and access to email, reside in the state in which the research was being conducted, and speak and read either English or Spanish. Recruitment, particularly in the beginning of the study, largely focused on parents residing in rural communities; ultimately, participants were drawn from both rural and metropolitan areas of the state in the Pacific Northwest region of the U.S.

Most parent participants identified as female (93%) and White, non-Hispanic/Latine (72%). The median annual family income bracket was $35,000–$49,999, substantially lower than the median household income in the U.S., which was approximately $75,000 in 2022, the year that recruitment began (43). Over two-thirds (69%) of the sample reported that their family received some government assistance in the past year, including Temporary Assistance for Needy Families (TANF), Supplemental Nutrition Assistance Program (SNAP) benefits, and/or Women, Infants, and Children (WIC) program benefits. The average age of the target children in the study at baseline was 3 years (*M = *43.3 months, *SD* = 14.2). Over forty percent of participants (43%) resided in a zip code designated as rural according to the Oregon Office of Rural Health geographic definitions (44). Demographic data are presented in Table 1.

**Table 1:** Participant demographic characteristics.

Parent demographic characteristics (*N*=346)	M (*SD*) or % endorsed
Child age (months)	43.30 (14.2)
Parent gender - female	92.7%
Parent race/ethnicity	
White, Non-Hispanic/Latine	71.6%
Hispanic/Latine	21.1%
American Indian/Alaska Native	5.6%
Asian	4.5%
Black/African American	4.2%
Native Hawaiian or Other Pacific Islander	2.0%
Parent age	33.70 (6.0)
Parent's highest education level	
Less than high school degree	7.8%
High school degree or GED	19.1%
Technical school	39.9%
Partial college	18.5%
Associate's degree	14.6%
Bachelor's degree	7.8%
Graduate training or degree	19.1%
Annual family income	
<$15,000	20.8%
$15,000-$34,999	21.3%
$35,000-$49,999	12.4%
$50,000-$74,999	17.4%
$75,000-$99,999	9.0%
>$100,000	14.6%
Family resides in rural area	43.0%
Adolescent-onset cannabis use	40.2%
Adolescent-onset of alcohol, heroin, or prescription drug misuse	42.7%
Current cannabis use (# of days used in past 30 days, for those reporting current use)	25.0%; 14.67 (12.3)

### 2.2 Procedures

Interested parents contacted the study team via phone call, email, or a form on the project's website. A staff member subsequently contacted the parent to explain the study, assess eligibility, and review informed consent if eligibility criteria were met. Following informed consent, parents completed baseline assessment questionnaires via phone interview or online survey, depending on parent preference. Next, they were randomized to the intervention or control condition. Parents in both the FCU-O and control groups completed a 3-month follow-up assessment. Parents in both groups were compensated $100 for each completed assessment. Parents randomized to the FCU-O did not receive any additional compensation for participating in either component (mobile app or telehealth coaching, see below) of the program.

#### 2.2.1 The FCU-O—parenting young children

The FCU-O—Parenting Young Children version used in the current study was adapted from the initial online iteration (45), which is grounded in the original FCU model (46). Like the original FCU and subsequent online version (38), the Parenting Young Children version of the FCU-O incorporates self-assessment and feedback on parenting skills and family management. The Parenting Young Children version was specifically developed for parents of toddler and preschool-age children who are at high risk of experiencing mental health and/or parenting challenges, including current or past substance use. Content was adapted from the Everyday Parenting curriculum (47), with additional focus on parent wellbeing and substance use psychoeducation using a harm reduction model. The FCU-O was adapted for early childhood with feedback from focus groups of parents and community providers (31). Content is divided into five modules: wellness and self-care, parenting and substance use, positive parenting, proactive parenting, and rules and consequences. The FCU-O is also available in Spanish. Parents had access to the app for one year after their enrollment in the program.

Parents in the intervention group were assigned to work with a telehealth family coach after completing the baseline assessment. The role of the family coach was to support parent's practice of skills learned through the app and work with the parent on applying the skills to their personal parenting goals and family situation. The family coach contacted the parent for an initial meeting to enroll them in and orient them to the FCU-O. Family coaches used motivational interviewing strategies to engage families, set goals, reinforce parents' existing strengths, and implement evidence-based parenting strategies. Family coaches were master's and doctoral-level clinicians trained to deliver the original FCU model with fidelity using the COACH (48), an observational coding system designed to measure interventionist fidelity to the core components of the FCU. Clinicians are rated on five dimensions, which include the following: Conceptually accurate and adherent to the model, Observant and responsive to family's needs, Active in structuring the session, Careful when teaching and providing feedback, and Helpful in building hope and motivation.

Telehealth coaching sessions were intended to flexibly correspond with parent's completion of each of the five app modules, with approximately five to six 20-min coaching sessions over a period of approximately three months. However, the frequency and duration of coaching sessions were tailored to parents' personal needs and goals. As such, parents may have continued to have follow-up sessions with their family coach after completing the 3-month follow-up assessment.

#### 2.2.2 Control condition

Parents randomly assigned to the control condition completed questionnaires at baseline and 3 months later. Approximately one year after completing the baseline assessment, the study team contacted parents in the waitlist control condition to offer them access to the FCU-O app program.

### 2.3 Measures

#### 2.3.1 Substance use

Caregivers completed a set of questions about their current and past use of substances, including alcohol, cannabis, and heroin, as well as their misuse of prescription opioids. Questions assessing substance use came from the PhenX Toolkit's Substance Abuse and Addiction (SAA) collection (49, 50). The SAA collection of the PhenX Toolkit is a publicly available catalog of valid and reliable measures to assess substance use, with the intention of increasing researchers' ability to harmonize data collection on substance use across studies. In the current study, adolescent-onset cannabis use was a dichotomous variable based on whether caregivers reported using cannabis regularly (i.e., at least once a month for 3 months in a row) beginning at age 19 or younger.

Adolescent-onset use of other substances (alcohol, heroin, prescription opioid misuse) was operationalized in the same manner. This was also a dichotomous variable in which if a parent endorsed regular use of either alcohol or heroin, or misuse of prescription opioids, they were categorized as having adolescent-onset use of other substances.

Current cannabis use was operationalized as a continuous variable based on caregivers' response to the question, “During the past three months, how many days did you use marijuana?” from the baseline questionnaire.

#### 2.3.2 Parent depressive symptoms

Caregivers completed the Patient Health Questionnaire-9 (PHQ-9; 51), a 9-item self-report, screening measure of depressive symptoms experienced during the past two weeks. Item responses range from 0 (“not at all”) to 3 (“nearly every day”). A mean score was calculated to represent total depressive symptoms at both baseline and 3-month follow-up assessments.

#### 2.3.3 Parent anxiety symptoms

Caregivers completed the Generalized Anxiety Disorder-7 (GAD-7; 52), a 7-item self-report, screening measure of anxiety symptoms experienced during the past two weeks. Like the PHQ-9, item responses range from 0 (“not at all”) to 3 (“nearly every day”). A mean score was calculated to represent total anxiety symptoms at both baseline and 3-month follow-up assessments.

#### 2.3.4 Parenting skills

The Parenting Young Children scale (PARYC; 53), a 21-item self-report measure, was used to measure parenting skills, including positive behavior support, proactive parenting, and limit-setting. Parents are asked to rate the frequency of their use of parenting skills in the past month on a scale from 1 (“not at all”) to 7 (“most of the time”). The PARYC contains three subscales: Supporting Positive Behavior (e.g., “reward your child when they did something well or showed a new skill”), Setting Limits (e.g., “explain what you wanted your child to do in clear and simple ways”), and Proactive Parenting (e.g., “avoid struggles with your child by giving clear choices”), each of which has 7 items. A mean score for each subscale was calculated for the baseline assessment.

#### 2.3.5 Parent pain interference

The PROMIS Pain Interference form (54) measures the consequences of pain experience on different life domains. In the current study, the PROMIS Adult Short Form v1.0—Pain Interference 4a, which contains four items, was used. Individuals were asked to rate on a 5-point scale (“not at all” to “very much”) the extent to which pain interfered with daily life (e.g., “in the past 7 days, how much did pain interfere with work around the home?”). Pain interference at baseline was included as a covariate in the current study given that cannabis is frequently used to treat chronic pain (55), and chronic pain frequently co-occurs with symptoms of anxiety and depression (56).

#### 2.3.6 Demographic characteristics

Information about parent demographic characteristics was collected through a demographic questionnaire administered at the baseline assessment. Family rurality status was determined based on whether their reported zip code was designated as rural vs. urban according to the Oregon Office of Rural Health geographic definitions (44). Family income was a categorical variable in which participants were asked to report the range in which their total family income fell (e.g., $35,000–$49,999).

### 2.4 Data analytic plan

Analyses were conducted using MPlus (57). All analyses included the following covariates: adolescent-onset use of substances other than cannabis (alcohol, heroin, or prescription medication misuse), family income, rural status, and current pain interference. Multivariate multiple regression analyses were used to test the effects of adolescent-onset and current cannabis use on caregiver mental health (anxiety and depression) and parenting skills (limit-setting, positive parenting, and proactive parenting. To test whether the FCU-O was particularly effective in improving symptoms of depression and/or anxiety for parents with a history of adolescent cannabis use, we created an interaction term for the intervention group × adolescent-onset cannabis use (both dichotomous variables). To probe the interaction, we plotted simple slopes for parents with and without a history of adolescent-onset cannabis use.

## 3 Results

### 3.1 Descriptive data and correlational analyses

Of the 356 parents included in the current study, 324 (91%) participated in the 3-month follow-up assessment. Forty percent of the sample reported adolescent-onset cannabis use (i.e., started using cannabis at least once a month for 3 months in a row starting at age 19 or younger) (see Table 1). Of those who reported ever having used cannabis at least once a month for 3 months in row, the mean age of initiating this frequency of usage was 17.7 years (*SD *= 5.3). About a third (31%) of participants reported depressive symptoms that fell within the clinical range (i.e., ≥10) on the PHQ-9 at the baseline assessment; 31% of participants indicated clinically significant anxiety symptoms (i.e., ≥10) on the GAD-7 at the baseline assessment. Approximately 25% of the sample reported having used cannabis at least once in the past 30 days; nationally, approximately 12% of adults aged 26 or older reported cannabis use in the past month (58).

The bivariate correlation analysis indicated that adolescent-onset cannabis use was significantly associated with rural residence, lower family income, adolescent-onset use of other substances, current cannabis use, and current symptoms of anxiety and depression (see Table 2). Current cannabis use was significantly associated with current symptoms of depression. Neither adolescent-onset nor current cannabis use was significantly associated with parenting skills.

**Table 2:** Correlations among study variables.

Variable	1	2	3	4	5	6	7	8	9	10
1. Family income	−									
2. Rural residence	−.07	−								
3. Pain interference	−.18*	−.01	−							
4. Adolescent-onset other substance use	.01	−.07	.02	−						
5. Adolescent-onset cannabis use	−.25*	.13*	.10	.49*	−					
6. Current cannabis use	−.19*	.10	.08	.11*	.35*	−				
7. Parent anxiety	−.14*	.14*	.27*	.07	.25*	.07	−			
8. Parent depression	−.18*	.13*	.40*	.02	.21*	.11*	.76*	−		
9. Parent limit-setting	.00	.05	.01	−.03	.00	.03	−.08	−.10	−	
10. Positive parenting	−.08	.14*	−.01	−.05	.07	.07	−.10	−.17*	.60*	−
11. Proactive parenting	−.07	.05	−.02	−.01	.03	−.01	−.05	−.08	.72*	.58*

All variables included in this table were measured at the baseline assessment.

*p < .05.

### 3.2 Effects of adolescent-onset and current cannabis use on caregiver mental health

In the multivariate multiple regression analysis testing the effects of both adolescent-onset and current cannabis use on parent depression and anxiety, including the covariates of family income, rurality, pain interference, and adolescent-onset use of other substances, there was a significant effect of adolescent-onset cannabis use on both parental depressive symptoms, *β* = .17, *p *< .01, and anxiety symptoms, *β* = .23, *p *< .01. There was no effect of current cannabis use on concurrent depressive or anxiety symptoms (see Table 3). Of the covariates, only caregivers' current pain interference was significantly associated with both depressive and anxiety symptoms. None of the other covariates were statistically significant.

**Table 3:** Effects of adolescent-onset and current cannabis use on parent mental health and parenting skills.

	Parent mental health				Parenting skills					
	Depression		Anxiety		Positive parenting		Proactive parenting		Limit-setting	
	β	*p*	β	*p*	β	*p*	β	*p*	β	*p*
Pain interference	.38*	.00	.25*	.00	.00	.99	−.03	.55	.06	.32
History of adolescent alcohol, opioid, or prescription opioid misuse	−.08	.15	−.05	.34	−.01	.90	.01	.94	.05	.40
Family income	−.07	.21	−.05	.35	−.06	.31	.00	.96	.09	.11
Rural status	.10*	.04	.09	.06	.10	.07	−.01	.83	.03	.64
Current cannabis use	.02	.70	−.04	.46	−.01	.89	.04	.50	−.03	.65
Adolescent-onset cannabis use	.17*	.00	.23*	.00	.01	.92	.03	.65	−.05	.43

*p < .05.

### 3.3 Effects of adolescent-onset and current cannabis use on caregiver parenting skills

In the multivariate multiple regression analysis testing the effects of both adolescent-onset and current cannabis use on parenting skills, including positive behavior support, limit-setting, and proactive parenting, neither adolescent-onset nor current cannabis use was significantly associated with any of the parenting skills tested (see Table 3). None of the covariates were significantly associated with parent-reported parenting skills, where parents living in rural areas reported significantly higher use of positive parenting, *β* = .11, *p *< .05.

### 3.4 Differential effects of the FCU-online for adolescent-onset cannabis users

We then tested whether the FCU-O was more effective in reducing symptoms of anxiety or depression for caregivers with a history of adolescent cannabis use. Covariates included all the aforementioned variables, as well as baseline anxiety or depressive symptoms to test change in symptoms from baseline to 3-month follow-up.

The main effect of the FCU-O on change in anxiety symptoms was not significant, *β* = .01, *p* = .88. The main effect of adolescent cannabis use was significant, *β* = .14, *p* = .05, indicating that parents who used cannabis in adolescence had increased anxiety from baseline to 3-month follow-up. The interaction term was also statistically significant, *β* = −.15, *p* = .03. When the interaction was probed using simple slopes analysis, it was determined that for caregivers with a history of adolescent cannabis use, the FCU-O was associated with a significant decrease in anxiety symptoms from baseline to 3-month follow-up, B = −1.93, *p *< .01. The effect of the FCU-O on anxiety symptoms for caregivers without a history of adolescent cannabis use was not significant, B = .09, *p = *.88. Of the covariates, pain interference significantly predicted anxiety at 3-month follow-up (*β* = .16, *p* < .01). Baseline anxiety also significantly predicted anxiety at 3-month follow-up (*β* = .59, *p* < .01). For the outcome of depressive symptoms, neither main effect, nor the interaction term, was statistically significant. In sum, we found that the FCU-O was more effective in reducing anxiety symptoms for parents who used cannabis regularly as an adolescent; this finding was not replicated with depressive symptoms.

## 4 Discussion

In sum, findings from the current study suggest that for parents of young children, adolescent-onset cannabis use, independent of current cannabis use, may be a risk factor for experiencing higher levels of depression and anxiety symptoms later in adulthood. Our findings also indicate that parents of young children with a history of adolescent-onset cannabis use may be more likely to experience reductions in anxiety following a supportive parenting intervention. Analyses did not provide support for the hypothesis that adolescent-onset cannabis use is associated with lower parenting skills.

Prior literature supports our finding that the initiation of regular cannabis use in adolescence may be a particular risk factor for experiencing symptoms of depression and anxiety in adulthood (23, 28). However, this is the first study to our knowledge to test the effects of both adolescent-onset cannabis use and current cannabis use in relation to depression and anxiety symptoms in parents of young children. Understanding factors that contribute to parents' mental health is critical due to robust evidence that symptoms of anxiety and depression compromise parenting skills (24, 25).

Although our primary focus in the current study was on adolescent-onset cannabis use, current cannabis use was also included in all models. Surprisingly, there was no effect of current cannabis use on symptoms of depression or anxiety. The bivariate correlation between current cannabis use and depressive symptoms was statistically significant, though small, and was no longer significant in the full regression model with adolescent-onset cannabis use and covariates. This finding is inconsistent with a great deal of prior studies indicating that cannabis use is associated with symptoms of anxiety and depression in the general adult population (59–62), with some prior work accounting for age at first use (61). The lack of associations is especially noteworthy given that participants in this study reported much higher rates of both current cannabis use (58) and depressive symptoms (63) than the national average, and recent research suggests that the association between depression and cannabis use among young people *increased* as a result of cannabis legalization (64).

We did not find any evidence that adolescent-onset or current cannabis use impacts parents' self-report of their own parenting skills in the areas of limit-setting, proactive parenting, or positive behavior support. One study found that only cannabis use disorder, not cannabis use without cannabis use disorder, was associated with lower levels of positive parenting in parents of adolescents (16). Most prior work has focused on parental cannabis use in relation to problematic or negative parenting behaviors including child abuse and neglect (13–15), so it could be that cannabis use is more likely to affect negative than positive parenting behaviors. No prior research has tested associations between parents' adolescent-onset cannabis use and parenting skills and behaviors. Future research should continue to explore this relationship.

Finally, we found that the FCU-O program was particularly beneficial in reducing anxiety for parents with adolescent-onset substance use. Paired with the result that parents with adolescent-onset cannabis use had higher levels of depression and anxiety at baseline, these findings suggest that although parents with adolescent-onset substance use may be more vulnerable to experiencing mental health challenges, they may also be more likely to respond to treatment. Given that parent psychopathological symptoms are associated with adolescent substance use (65), this finding suggests that improving parental anxiety through a parenting support program such as the FCU-O provides an important step toward reducing multigenerational risk for substance use and mental health challenges. In particular, reducing parent anxiety may improve parental responsivity towards their child (66) which supports children's developing emotion regulation skills (67), a protective factor for adolescent substance use (68).

It is unclear why this pattern of results was found only for parent anxiety, not depression. Interestingly, using the same sample as that of the current study, researchers found that random assignment to the FCU-O group was associated with reductions in parents' depression, but not anxiety (see text footnote 1). To fully understand and interpret this finding, it would be important to better understand what factors are associated with adolescent-onset cannabis use that could explain the effects. For example, it could be that a longstanding history of mental health challenges beginning in adolescence, which was not measured in the current study, may be driving the effect.

This study has several limitations that are important for the interpretation and generalizability of its findings. Adolescent cannabis and other substance use was reported retrospectively, which can be inconsistent with adolescents' reporting of their cannabis use in real time (69). Given stigma related to parental substance use in the healthcare system (70) and society at large, there may also be limitations in the validity of self-reported substance use data for parents of young children (71). Additionally, other than age of initiation of substance use, we did not collect any other data specifically focusing on parents' adolescence. As mentioned above, it could be that other, unmeasured variables, such as adolescent-onset depression or anxiety, are driving the effects found in the current study. A large, prospective study that begins in adolescence and collects data on substance use and mental health through the transition to parenthood would be ideal for understanding inter-relationships among cannabis use, mental health, and parenting. Other limitations include that parenting skills were measured using parent self-report. Given prior research that parents perceive cannabis to improve their parenting skills (72), it may be useful for future work to include observational measures of parenting behavior. In addition, participants in this study were predominantly White and non-Hispanic/Latine, which is reflective of the demographics of the state in which the study was conducted, particularly in rural areas. Finally, our investigation into whether the FCU-O is particularly effective in improving mental health symptoms for parents with adolescent-onset cannabis use was exploratory, given the minimal extant literature on how adolescent substance use may impact future parenting skills and response to a parenting intervention. Future research is necessary to replicate this finding.

In conclusion, findings from the current study indicate that the onset of regular cannabis use in adolescence is associated with increased symptoms of anxiety and depression in parents of young children, as well as a greater *reduction* in symptoms of anxiety following a digital parenting intervention, the FCU-O. There was no indication that current or past cannabis use was associated with self-reported parenting skills. Taken together, this study provides important avenues for future research, including the importance of testing effects of both current and adolescent cannabis use on positive parenting skills, and exploring the differential effectiveness of parenting interventions for current and adolescent cannabis users.

## Data Availability

The raw data supporting the conclusions of this article will be made available by the authors, without undue reservation.

## Footnotes

^1^Hails KA, McWhirter AC, Sileci ACB, Stormshak EA. Family check-up online effects on parenting and parent wellbeing in families of toddler to preschool-age children.
